# Structural factors associated with an increased risk of HIV and sexually transmitted infection transmission among street-involved youth

**DOI:** 10.1186/1471-2458-9-7

**Published:** 2009-01-09

**Authors:** Brandon DL Marshall, Thomas Kerr, Jean A Shoveller, Julio SG Montaner, Evan Wood

**Affiliations:** 1 British Columbia Centre for Excellence in HIV/AIDS, St. Paul's Hospital, 608 – 1081 Burrard Street, Vancouver, BC, V6Z 1Y6, Canada; 2 School of Population and Public Health, University of British Columbia, 5804 Fairview Avenue, Vancouver, BC, V6T 1Z3, Canada; 3 Department of Medicine, University of British Columbia, 608 – 1081 Burrard Street, Vancouver, BC, V6Z 1Y6, Canada

## Abstract

**Background:**

The prevalence of HIV and sexually transmitted infections (STIs) among street-involved youth greatly exceed that of the general adolescent population; however, little is known regarding the structural factors that influence disease transmission risk among this population.

**Methods:**

Between September 2005 and October 2006, 529 street-involved youth were enroled in a prospective cohort known as the At Risk Youth Study (ARYS). We examined structural factors associated with number of sex partners using quasi-Poisson regression and consistent condom use using logistic regression.

**Results:**

At baseline, 415 (78.4%) were sexually active, of whom 253 (61.0%) reported multiple sex partners and 288 (69.4%) reported inconsistent condom use in the past six months. In multivariate analysis, self-reported barriers to health services were inversely associated with consistent condom use (adjusted odds ratio [aOR] = 0.52, 95%CI: 0.25 – 1.07). Structural factors that were associated with greater numbers of sex partners included homelessness (adjusted incidence rate ratio [aIRR] = 1.54, 95%CI: 1.11 – 2.14) and having an area restriction that affects access to services (aIRR = 2.32, 95%CI: 1.28 – 4.18). Being searched or detained by the police was significant for males (aIRR = 1.36, 95%CI: 1.02 – 1.81).

**Conclusion:**

Although limited by its cross-sectional design, our study found several structural factors amenable to policy-level interventions independently associated with sexual risk behaviours. These findings imply that the criminalization and displacement of street-involved youth may increase the likelihood that youth will engage in sexual risk behaviours and exacerbate the negative impact of resultant health outcomes. Moreover, our findings indicate that environmental-structural interventions may help to reduce the burden of these diseases among street youth in urban settings.

## Background

Structural factors, defined as the economic, social, policy, and organizational environments that "structure" the context in which risk production occurs [1], are increasingly recognised as important determinants in the acquisition, transmission, and prevalence of HIV disease [2]. In recent years, extensive research has examined the structural factors that produce and re-produce HIV risk among high prevalence populations, including injection drug users (IDU) and sex workers [3,4]. Homeless and street-involved adolescents have also been recognised as a marginalised population with unique exposures to structural environments that increase the likelihood of sustained and elevated disease burden; however, these factors remain poorly understood [5].

In Canada and the United States, it is estimated that between 4 and 7 percent of youth between the ages of 14 and 26 are absolutely, periodically, or temporarily without access to safe and stable shelter [6,7]. Homeless and street-involved youth are known to be at a significantly increased risk for a wide range of adverse health outcomes [8]. Of considerable public health concern is the high prevalence of HIV and sexually transmitted infections (STIs) among these populations. In urban centres in Canada, the prevalence of HIV among street-involved youth is approximately 2 percent [9,10], while the prevalence of Chlamydia has been estimated to be between 7 and 11 percent [11,12]. Similar rates have been observed in the United States [13,14].

Street-involved youth engage in a greater number of sexual risk behaviours than their non-homeless peers [15]. The vast majority is sexually active, and among those who do engage in sexual intercourse, inconsistent condom use is common [16,17]. Street-involved youth are also more likely to have multiple and concurrent sex partners [18,19]. Of further concern is that approximately one quarter of street youth have engaged in survival sex (i.e., sex in exchange for money, shelter, food or drugs) [20]. Among youth who are coerced or manipulated into survival sex, sexual victimization and abuse are common [21].

Research that has attempted to elucidate the underlying reasons for increased engagement in sexual risk behaviour among street-involved youth has continued to rely predominantly on individual level risk factor analyses [22]. However, a growing body of literature has demonstrated that a focus on individual level characteristics (e.g., childhood abuse, depression, knowledge) fails to acknowledge the social structural factors that shape and determine the context in which sexual risk behaviour takes place [3,23]. Furthermore, it is increasingly recognised that structural factors, including economic inequities, laws, policies, and systemic discrimination, are better overall predictors of population level HIV and STI prevalence [24]. Given these methodological challenges and concerns, we sought to determine whether structural factors are associated with increased engagement in sexual risk behaviour among a community-recruited cohort of street-involved youth.

## Methods

The At Risk Youth Study (ARYS) is a prospective cohort of homeless and street-involved youth in Vancouver, Canada that has been described in detail previously [25]. Briefly, participants were recruited through snowball sampling and extensive street-based outreach. Persons were eligible for the study if they were 14 to 26 years of age, had used illicit drugs other than or in addition to marijuana in the past 30 days, and provided informed consent. At baseline and semi-annually, participants complete an interviewer-administered questionnaire and provide blood samples for HIV and hepatitis C (HCV) serology. The questionnaire elicits demographic data and information regarding injection and non-injection drug use, HIV risk behaviours, addiction treatment experience, encounters with police and security guards, health service utilization, and sexual activity. All participants receive a monetary stipend of $20 CDN after each visit. The study has been approved by the University of British Columbia/Providence Health Care Research Ethics Board.

All participants who completed a baseline survey between September 2005 and October 2006 were included in this study. Since data from just one follow-up period was available at the time of study conception, only information collected at baseline was included in these analyses. We examined as our primary outcomes two sexual risk behaviours that together play key roles in determining the sexual transmission of HIV and STIs: 1) number of sexual partners, and 2) condom use during vaginal and anal intercourse. Participants were asked to report how many different male and female partners they had engaged in sexual activities with in the past 6 months, excluding those with whom they had engaged in sex for money, shelter, food, or drugs (i.e., sex trade work). Specifically, the total number of partners was obtained by adding responses to the questions: "Could you give me a precise number of male/female partners you had in the past 6 months?". Participants could report any set of positive integer values; thus, the variable was coded as continuous in bivariate and multivariate analyses. The resulting distribution was positively skewed, with a median of 1.0 (interquartile range: 0–3), a mean of 3.2 (standard deviation: 5.6), and a range of 0–55. For both same and opposite sex partnerships, participants were also asked to report how often a condom was used during vaginal and/or anal intercourse. Possible responses included: always (100%), regularly (50% to 99%), occasionally (1% to 49%), and never (0%). To be consistent with previous studies of condom use among street-involved youth populations [26], this variable was dichotomised into "consistent" (i.e., always) and "inconsistent" (i.e., regularly, occasionally, or never) condom use. Participants who reported more than one type of sexual activity and who reported discordant condom use patterns were coded as inconsistent condom users.

The primary variables of interest in this study were a set of variables addressing structural factors that were hypothesised to shape the context in which street youth sexual risk behaviour is produced. We defined: "homeless" as any participant who reported being homeless in the past six months; "barriers to health or harm reduction services" as being in need of but unable to obtain health or harm reduction services (e.g., doctor, nurse, clinic, dentist, optometrist, or needle exchange); "jacked up" as being stopped, searched or detained by the police; "warrants" as currently having a warrant or area restriction that affects access to needle exchange programs (NEP) or other services; "unable to access treatment" as trying to access an alcohol or drug treatment program but being unable to; and "assault from police/security guards" as experiencing a physical interaction with police or security guards resulting in bruises, scratches, etc. All variables except for "warrants" refer to behaviours and events occurring in the past six months since the date of the interview. Other independent variables included a broad range of sociodemographic, individual level, drug-related, and social factors, chosen based on their known or *a priori *status as risk factors for one or both sexual behaviour outcomes. Sociodemographic variables that were examined included: age, sex (female vs. male), Aboriginal ethnicity (yes vs. no) and sexual orientation (lesbian, gay bisexual, transgendered/transsexual [LGBTT] vs. heterosexual). Other individual level factors that were examined included: engaging in anal intercourse in the past six months, depression (defined using the Centre for Epidemiologic Studies Depression [CES-D] scale), and the self-efficacy for limiting HIV risk behaviours (LHRB) scale. The CES-D has been shown to have high levels of internal consistency and reliability among groups of adolescents [27]. The presence of depressive symptoms was evaluated using a well-defined cut-off (CES-D ≥ 16 [yes] versus CES-D < 16 [no]). The self-efficacy for LHRB scale is a validated instrument found to have high levels of consistency among at-risk youth [28]. Responses were dichotomised into "high" versus "low" self-efficacy for LHRB based on the sample median. Social and drug-related factors that were examined included: relationship status (single or casually dating vs. regular partner or married), childhood sexual abuse, drug dealing, alcohol dependence, crack use, cocaine use, heroin use, crystal methamphetamine use, injection drug use, syringe sharing, and binge drug use (yes vs. no). All drug use variables refer to behaviours occurring in the past six months and include both injection and non-injection routes of consumption. To be consistent with our previous work, "syringe sharing" was defined as lending or borrowing a syringe that had been used by someone else, and "binge drug use" was defined as the self-reported consumption of drugs (injection or non-injection) more often than usual [29]. Finally, alcohol dependence was measured using the Perceived-Benefit-of-Drinking Scale (PBDS), a validated true/false instrument that assesses drinking behaviours among adolescents [30].

Initially, we examined bivariate associations between each independent variable and each sexual risk behaviour outcome. Given that the precise number of recent sexual partners was obtained for each participant, we used a Poisson-type regression to estimate the unadjusted incidence rate ratio (IRR) and 95% confidence interval (95% CI) associated with each explanatory variable. Since the distribution of recent sex partners was highly skewed, we used a log-linear quasi-Poisson regression to account for overdispersion in the data. To examine the bivariate associations between each independent variable and consistent condom use, we used the Pearson χ^2 ^test. Fisher's exact test was used when one or more of the cells contained values less than or equal to 5. Since sexual risk behaviour profiles among street-involved youth are observed to be moderated by gender [31,32], we also assessed each structural variable for possible interaction with sex. If a statistically significant interaction effect was observed, the coefficients corresponding to the main and interaction terms were combined to construct IRR estimates corresponding to each sex. The overall significance of the main and interaction effect was assessed using the likelihood ratio test.

Since research among populations of IDU and street-based sex workers has demonstrated that policies and laws promoting the displacement and criminalization of marginalised persons are associated with sexual- and injection-related HIV risk production [3,4,33], we chose to focus our analysis on structural variables that address these issues and thus may potentially shape the production of sexual risk-taking behaviour among street-involved youth. In order to account for potential confounding, we used an *a priori *defined bivariate cut-off of *p *< 0.10 as the criterion for inclusion of variables into multivariate analyses. Each independent variable was included as a potential explanatory factor when not used as the primary outcome of interest. All statistical analyses were conducted using S-PLUS software version 8.0. All reported *p*-values are two-sided.

## Results

A total of 529 participants completed an interview between September 1, 2005 and October 31, 2006, of whom 159 (30.1%) were female, 127 (24.0%) were of Aboriginal ethnicity, and 69 (13.0%) self-identified as LGBTT. The majority, 415 (78.4%), reported engaging in voluntary sexual activity in the past six months. Of these participants, 288 (69.4%) reported inconsistent condom use and 253 (61.0%) reported multiple sex partners. Of the entire sample, the median number of sex partners in the past six months was 1 (interquartile range [IQR]: 1 – 3; range: 0 – 55).

The results of the bivariate quasi-Poisson analyses are shown in Table 1. Structural variables that were positively associated with number of recent sex partners included homelessness (incidence rate ratio [IRR] = 1.87, 95% confidence interval [95%CI]: 1.24 – 2.82) and having a warrant or area restriction that affects access to services (IRR = 2.51, 95%CI: 1.21 – 5.18). Statistically significant interaction effects were observed for both "jacked up" and "barriers to health or harm reduction services" variables. The former was positively associated with number of recent sex partners for males (IRR = 1.53, 95%CI: 1.07 – 2.18), while the latter was marginally significant for females (IRR = 1.92, 95%CI: 0.97 – 3.79). Barriers to accessing health or harm reduction services (odds ratio [OR] = 0.53, 95% CI: 0.28 – 1.00) was the only structural factor associated with consistent condom use in bivariate analysis (see Table 2). The results of the multivariate analyses modelling number of recent sex partners and consistent condom use are shown in Tables 1 and 3, respectively. Homelessness (adjusted incidence rate ratio [aIRR] = 1.54, 95% CI: 1.11 – 2.14) and having a warrant or area restriction that affects access to services (aIRR = 2.32, 95% CI: 1.28 – 4.18) were positively and independently associated with number of recent sex partners. Furthermore, the overall contributions (main and interaction effect) of both "jacked up" and "barriers to health or harm reduction services" to the final model were highly significant (*p *< 0.001 for both variables). For males, being jacked up by the police was positively associated with number of recent sex partners (aIRR = 1.36, 95% CI: 1.02 – 1.81), while barriers to health or harm reduction services was marginally significant for females (aIRR = 1.76, 95% CI: 0.98 – 3.15). In multivariate logistic regression analysis, barriers to accessing health or harm reduction services was marginally and inversely associated with consistent condom use (adjusted odds ratio [aOR] = 0.52, 95% CI: 0.25 – 1.07); no significant interaction with sex was observed.

**Table 1:** Factors associated with number of sex partners among a cohort of street-involved youth (n = 529).

	**Unadjusted Incidence Rate Ratio (IRR)**			**Adjusted Incidence Rate Ratio (aIRR)**		
**Characteristic**	**IRR**	**(95% CI)**	***p *– value**	**aIRR**	**(95% CI)**	***p *– value**
**Age **(per year older)	0.98	(0.93 – 1.04)	0.566			
**Sex **(female vs. male)	0.80	(0.57 – 1.13)	0.198			
**Aboriginal ethnicity **(yes vs. no)	0.84	(0.58 – 1.22)	0.366			
**Sexual orientation **(LGBTT^a ^vs. heterosexual)	1.90	(1.37 – 2.63)	< 0.001	1.58	(1.16 – 2.16)	0.004
**Relationship **(single vs. partner)	1.79	(1.19 – 2.69)	0.005	1.44	(1.04 – 2.00)	0.028
**Depression^b ^**(yes vs. no)	1.14	(0.84 – 1.56)	0.402			
**Self-Efficacy LHRB^c ^**(low vs. high)	1.55	(1.14 – 2.13)	0.006	1.41	(1.10 – 1.81)	0.007
**Condom use^† ^**(consistent vs. inconsistent)	0.86	(0.62 – 1.20)	0.380			
**Anal intercourse^† ^**(yes vs. no)	2.52	(1.83 – 3.48)	< 0.001	2.01	(1.51 – 2.69)	< 0.001
**Sexual abuse^‡ ^**(yes vs. no)	1.67	(1.25 – 2.24)	< 0.001	1.40	(1.08 – 1.83)	0.011
**Drug dealing^† ^**(yes vs. no)	1.30	(0.95 – 1.77)	0.104			
**Alcohol dependence **(yes vs. no)	1.32	(0.97 – 1.79)	0.073	1.05	(0.82 – 1.35)	0.711
**Crack use^† ^**(yes vs. no)	1.45	(1.07 – 1.98)	0.018	1.20	(0.88 – 1.64)	0.249
**Cocaine use^† ^**(yes vs. no)	1.62	(1.20 – 2.19)	0.002	1.63	(1.28 – 2.08)	< 0.001
**Heroin use^† ^**(yes vs. no)	0.99	(0.72 – 1.37)	0.952			
**Crystal meth use^† ^**(yes vs. no)	1.07	(0.79 – 1.44)	0.671			
**Injection drug use^† ^**(yes vs. no)	1.01	(0.73 – 1.40)	0.944			
**Sharing syringes^† ^**(yes vs. no)	1.17	(0.72 – 1.91)	0.521			
**Binge drug use^† ^**(yes vs. no)	1.35	(1.00 – 1.83)	0.047	0.94	(0.71 – 1.26)	0.681
**Homelessness^† ^**(yes vs. no)	1.87	(1.24 – 2.82)	0.003	1.54	(1.11 – 2.14)	0.011
**Barriers to health/HR^d ^services^† ^**(yes vs. no)			< 0.001*			< 0.001*
**Male**	0.97	(0.62 – 1.51)	0.889	0.82	(0.57 – 1.16)	0.259
**Female**	1.92	(0.97 – 3.79)	0.061	1.76	(0.98 – 3.15)	0.058
**Jacked up^† ^**(yes vs. no)			< 0.001*			< 0.001*
**Male**	1.53	(1.07 – 2.18)	0.020	1.36	(1.02 – 1.81)	0.034
**Female**	1.15	(0.62 – 2.10)	0.661	0.85	(0.51 – 1.41)	0.526
**Warrants **(yes vs. no)	2.51	(1.21 – 5.18)	0.007	2.32	(1.28 – 4.18)	0.005
**Unable to access treatment^† ^**(yes vs. no)	1.14	(0.74 – 1.78)	0.545			
**Assault from police/guards^† ^**(yes vs. no)	1.12	(0.79 – 1.61)	0.500			

Note: a – LGBTT denotes lesbian, gay, bisexual or transgendered/transsexual; b – CES-D standard cut-off score of 16 or greater;

c – denotes self-efficacy for limiting HIV risk behaviours scale; d – HR denotes harm reduction; † – refers to activities in the past 6 months; ‡ – refers to lifetime history; * – overall *p*-value for main and interaction effect

**Table 2:** Factors associated with consistent condom use among a cohort of street-involved youth (n = 415).

**Characteristic**	**Consistent** **n (%)** **n = 127**	**Inconsistent** **n (%)** **n = 288**	**Odds Ratio** **(95% CI)**	***p *– value**
**Age^¶^**				
< 22	61 (48.0)	162 (56.3)	0.72 (0.47 – 1.09)	0.150
≥ 22	66 (52.0)	126 (43.7)		
**Sex**				
Female	36 (28.6)	95 (33.1)	0.81 (0.51 – 1.28)	0.426
Male	90 (71.4)	192 (66.9)		
**Aboriginal ethnicity**				
Yes	37 (29.1)	67 (23.3)	1.36 (0.85 – 2.17)	0.251
No	90 (70.9)	221 (76.7)		
**Sexual orientation**				
LGBTT^a^	9 (7.1)	44 (15.3)	0.42 (0.20 – 0.90)	0.033
Heterosexual	117 (92.9)	243 (84.7)		
**Relationship status**				
Single/Dating	105 (84.0)	193 (67.7)	2.50 (1.46 – 4.30)	0.001
Regular Partner	20 (16.0)	92 (32.3)		
**Depression^b^**				
Yes	63 (51.2)	153 (55.2)	0.98 (0.64 – 1.49)	0.526
No	60 (48.8)	124 (44.8)		
**Self Efficacy LHRB^c^**				
Low	46 (36.8)	129 (46.4)	0.67 (0.44 – 1.04)	0.091
High	79 (63.2)	149 (53.6)		
**Number of sex partners^†^**				
> 1	79 (62.2)	165 (57.3)	1.23 (0.80 – 1.88)	0.407
≤ 1	48 (37.8)	123 (42.7)		
**Anal intercourse^†^**				
Yes	12 (9.8)	59 (20.9)	0.41 (0.21 – 0.79)	0.010
No	111 (90.2)	223 (79.1)		
**Sexual abuse^‡^**				
Yes	33 (26.6)	79 (27.7)	0.95 (0.59 – 1.52)	0.912
No	91 (73.4)	206 (72.3)		
**Drug dealing^†^**				
Yes	71 (55.9)	175 (60.8)	0.82 (0.54 – 1.25)	0.412
No	56 (44.1)	113 (39.2)		
**Alcohol dependence**				
Yes	56 (47.1)	154 (55.8)	0.70 (0.46 – 1.09)	0.137
No	63 (52.9)	122 (44.2)		
**Crack use^†^**				
Yes	67 (52.8)	174 (60.4)	0.73 (0.48 – 1.12)	0.177
No	60 (47.2)	114 (39.6)		
**Cocaine use^†^**				
Yes	58 (45.7)	137 (47.6)	0.93 (0.61 – 1.41)	0.802
No	69 (54.3)	151 (52.4)		
**Heroin use^†^**				
Yes	43 (33.9)	93 (32.3)	1.07 (0.69 – 1.67)	0.842
No	84 (66.1)	195 (67.7)		
**Crystal meth use^†^**				
Yes	52 (40.9)	146 (50.7)	0.67 (0.44 – 1.03)	0.084
No	75 (59.1)	142 (49.3)		
**Injection drug use^†^**				
Yes	35 (27.6)	84 (29.2)	0.92 (0.58 – 1.47)	0.829
No	92 (72.4)	204 (70.8)		
**Syringe sharing^†^**				
Yes	7 (5.5)	32 (11.1)	0.47 (0.20 – 1.09)	0.105
No	120 (94.5)	256 (88.9)		
**Binge drug use^†^**				
Yes	49 (39.8)	139 (49.5)	0.68 (0.44 – 1.04)	0.094
No	74 (60.2)	142 (50.5)		
**Homeless^†^**				
Yes	91 (71.7)	227 (78.8)	0.68 (0.42 – 1.10)	0.143
No	36 (28.3)	61 (21.2)		
**Barriers to health/HR^d ^services^†^**				
Yes	14 (11.0)	54 (18.9)	0.53 (0.28 – 1.00)	0.065
No	113 (89.0)	232 (81.1)		
**Jacked up^†^**				
Yes	55 (44.4)	135 (47.5)	0.88 (0.58 – 1.35)	0.628
No	69 (55.6)	149 (52.5)		
**Warrants**				
Yes	4 (3.2)	5 (1.8)	1.82 (0.48 – 6.92)	0.492
No	121 (96.8)	275 (98.2)		
**Unable to access treatment^†^**				
Yes	13 (10.2)	37 (12.9)	0.77 (0.39 – 1.51)	0.548
No	114 (89.8)	250 (87.1)		
**Assault from police/guards^†^**				
Yes	27 (21.8)	67 (23.7)	0.90 (0.54 – 1.49)	0.771
No	97 (78.2)	216 (76.3)		

Note: a – LGBTT denotes lesbian, gay, bisexual or transgendered/transsexual; b – CES-D standard cut-off score of 16 or greater; c – denotes self-efficacy for limiting HIV risk behaviours scale;

d – HR denotes harm reduction; ¶ – dichotomisation based on sample median; † – refers to activities in the past 6 months; ‡ – refers to lifetime history.

**Table 3:** Logistic regression analysis of factors associated with consistent condom use among a cohort of street-involved youth (n = 415).

**Variable**	**Adjusted Odds Ratio (AOR)**	**95% Confidence Interval (95% CI)**	***p *– value**
**Sexual orientation**			
(LGBTT^a ^vs. heterosexual)	0.38	(0.15 – 0.97)	0.044
**Relationship status**			
(single/dating vs. regular)	2.82	(1.59 – 5.01)	< 0.001
**Self-Efficacy LHRB^b^**			
(low vs. high)	0.66	(0.41 – 1.07)	0.091
**Anal intercourse^†^**			
(yes vs. no)	0.61	(0.30 – 1.24)	0.173
**Crystal meth use^†^**			
(yes vs. no)	0.74	(0.47 – 1.19)	0.217
**Binge drug use^†^**			
(yes vs. no)	0.67	(0.42 – 1.08)	0.098
**Barriers to health/HR^c ^services^†^**			
(yes vs. no)	0.52	(0.25 – 1.07)	0.074

Note: a – LGBTT denotes lesbian, gay, bisexual or transgendered/transsexual; b – denotes self-efficacy for limiting HIV risk behaviours scale; c – HR denotes harm reduction; † – refers to activities in the past 6 months.

## Discussion

These findings reveal high rates of inconsistent condom use and multiple sexual partnerships among a cohort of street-involved youth in Vancouver, Canada. Given that these behaviours describe two parameters which partially determine the population level transmission dynamics of HIV and STIs, we conclude that the continued propagation of these diseases among this population is likely. Our results also suggest that structural factors may play a role in driving risk behaviours that increase the likelihood of HIV and STI transmission. Further, the impact of structural factors on the sexual risk behaviours of street-involved youth appear to be moderated by gender, leading us to conclude that the intersection of structural determinants with gender and sexual inequities may promote the production of HIV risk within this population.

Having a warrant or area restriction that affects access to NEPs or other services was the strongest correlate of number of recent sex partners, even after adjustment for potential confounders such as homelessness. Furthermore, being "jacked up" by the police was independently associated with number of recent sex partners among males in our sample. These findings suggest that enforcement-based policies and practices which result in the criminalization of street youth activity may be a contributing factor in the production of HIV and STI risk among these populations. While few studies have characterised the potential impact of policing and enforcement policies on HIV and STI transmission among street youth, several authors have argued that street-level law enforcement promotes HIV risk behaviour among older populations including IDU who consume drugs in public spaces. Ethnographic research among IDU has shown that having outstanding warrants exacerbates the health and safety concerns associated with public injection due to fears of being arrested by police [34]. Specific enforcement practices may also impact the spread of HIV and STIs through more direct mechanisms. For example, the separation of sex partners due to the removal or displacement of individuals from normative structural environments has been theorised to increase the likelihood of new discordant sexual partnerships and riskier sexual behaviours [24]. Our results provide quantitative evidence to support these hypotheses and also indicate that more research is required to examine how police and other authority figures interact with street-involved youth in such a way that augments the production of HIV risk.

Our finding that individuals who have experienced barriers to health and harm reduction services were half as likely to report consistent condom use is worrisome. Judgmental policies and procedures, a failure to adhere to sex-positive principles, and a lack of systems that discourage heterosexist cultures have all been recognised as structural barriers that prevent street-involved youth from accessing services that sell or distribute condoms [35,36]. It is important to note that our findings regarding barriers to health or harm reduction services must be interpreted cautiously, as the associations between service barriers and both sexual risk behaviour outcomes achieved only marginal statistical significance. However, these results do provide further evidence for the hypothesised association between barriers to health care and harm reduction services and increased HIV and STI rates within street youth communities [37]. Future studies should seek to examine how specific mechanisms or barriers (e.g., stigma, inadequate coverage, inappropriateness of services) influence the accessibility and use of HIV/STI programs and resources for this population.

The other two factors that were associated with both sexual risk behaviour outcomes included sexual orientation and relationship status. Our finding that LGBTT-identified individuals are more likely to have multiple sex partners and less likely to report consistent condom use is worrisome and suggests that the development of interventions sensitive to diverse sexualities and orientations is urgently required. Furthermore, these results corroborate other research in the United States demonstrating that LGBTT homeless youth are at increased risk for a host of negative sexual health outcomes [35,38]. Our finding that homeless youth who are single or casually dating have more sexual partners but higher rates of condom use is also consistent with other studies demonstrating that relationship status is a strong determinant of sexual risk behaviour among youth at high risk for HIV [39,40]. Sexual abuse and drug use including cocaine consumption, both significantly associated with number of sex partners in our study, have also been associated with increased numbers of sex partners in other studies of homeless adolescents [41,42].

This study has a number of important implications for policies, programmes, and interventions that attempt to reduce population level burden of HIV and STI among young street-involved communities. We have shown that the displacement of street youth and the regulation of their behaviour through law enforcement strategies and other legal practices are independently associated with behaviours that increase the likelihood of HIV and STI transmission. Therefore, socio-legal reforms that de-emphasise enforcement-based policies and incorporate health or harm reduction frameworks may be effective at reducing HIV and STI incidence in the future. For example, policy and legal reforms that promote the health and safety of street-based sex workers have been shown to be effective at reducing HIV vulnerability among these populations [43]. Consistent with other studies [42,44], our results also indicate that homelessness may be an important driver of HIV and STI transmission. Interventions and public health programmes may seek to target youth who are homeless and deeply entrenched within the street culture and economy, and may benefit from incorporating youth-friendly, sex-positive policies and practices that reduce social-structural barriers to traditional health care environments. For example, street-based STI testing that is incorporated within pre-existing outreach services has been shown to be highly effective at reducing the structural barriers associated with traditional hospital or clinic settings [37].

This study has a number of sampling and methodological limitations. It is important to note that, due to the cross-sectional nature of the study design, these results are correlational and therefore no inferences can be made with respect to causation. It is possible that the constellation of structural factors observed in this study may simply cluster among youth who are more likely to engage in sexual risk behaviours for other unmeasured reasons. However, it is noteworthy that since all of the structural variables significant in bivariate analyses remained significant in the multivariate models, they do not appear to be confounded by each other. Future research using longitudinal study designs are required to corroborate these findings. Secondly, although extensive snowball and street-based outreach was used in an attempt to maximise the representativeness of our sample, we are unable to generalise our findings to other settings with different structural environments. Thirdly, the low sample size across several covariates resulted in wide confidence intervals that may have reduced our ability to observe small but significant effects. Therefore, we encourage the cautious interpretation of marginally significant results. Lastly, it is also possible that socially desirable reporting resulted in an under-estimate of stigmatised behaviours such as anal intercourse and inconsistent condom use, particularly with casual sex partners. However, we have no reason to suspect that differential reporting of these behaviours occurred between those who reported structural barriers and those that did not.

## Conclusion

We have shown that structural factors, in particular those that correspond to the displacement, regulation, and criminalization of street youth activity, are correlated with behaviours which increase the likelihood for HIV and STI transmission. Furthermore, street-involved youth who report barriers to traditional health or harm reduction services are more likely to engage in sexual risk behaviours that put them at an increased risk for the acquisition and transmission of these diseases. Structural factors remained associated with the drivers of HIV and STI transmission independently of individual, social, and drug-related characteristics; therefore, structural interventions that incorporate youth-friendly, accessible, health-based policies and practices may be effective at improving population level sexual health outcomes. These findings support the need for innovative interventions including legal reforms, non-coercive policing practices, and street-based outreach and sexual health services to reduce the prevalence of HIV and other STIs among marginalised youth populations in the future.

## Competing interests

The authors declare that they have no competing interests.

## Authors' contributions

EW had full access to all of the data and takes responsibility for the integrity of the results and the accuracy of the statistical analysis. BM, TK, and JS conceived the study concept and design and BM was responsible for the composition of the manuscript. The statistical analysis was conducted by BM, and the interpretation of the results was performed by BM, TK, JS, JM, and EW. The manuscript was edited and revised by BM, TK, JS, JM and TK. All authors read and approved the final manuscript

## Funding

The study was supported by the US National Institutes of Health (R01 DA011591) and Canadian Institutes of Health Research (HHP-67262 and RRA-79918). Thomas Kerr is supported by a New Investigator Award from CIHR and a Scholar Award from the Michael Smith Foundation for Health Research (MSFHR). Jean Shoveller is supported by a Senior Scholar Award from MSFHR and a Public Health Chair in Improving Youth Sexual Health from CIHR. Brandon Marshall is supported by a Canada Graduate Scholarship from CIHR and a Junior Graduate Trainee Award from MSFHR.

## Pre-publication history

The pre-publication history for this paper can be accessed here:


